# Community Cancer Rates in Massachusetts, New Hampshire, and Rhode Island: Findings From Healthy Aging Data Reports

**DOI:** 10.1093/geroni/igaa057.358

**Published:** 2020-12-16

**Authors:** Shuangshuang Wang, Chae Man Lee, Bon Kim, Nina Silverstein, Frank Porell, Beth Dugan

**Affiliations:** 1 Shandong University, Jinan, Shandong, China; 2 University of Massachusetts Boston, Boston, United States; 3 University of Massachusetts Boston, Boston, Massachusetts, United States; 4 University of Massachusetts Boston, Needham, Massachusetts, United States

## Abstract

Cancer is one major health condition that affect people’s later life quality, which could be intervened from the community level. This study compares rates of lung cancer, colon cancer, breast cancer (in women), and prostate cancer (in men) among adults 65+ in 3 New England states (MA, NH, and RI). Data were from the Healthy Aging Data Report (see www.healthyagingdatareports.org), which reported on 150+ health indicators at the local community and state level. Data sources were the Current Medicare Beneficiary Summary File (years) and the American Community Survey (years). Small area estimation techniques were used to calculate age-sex adjusted community rates. Average state rates of cancers (range) are: Lung cancer MA 2.1 (1.0 – 4.4) NH 1.6 (0.9 – 2.9) RI 2.1 (1.3 – 2.9); Colon cancer MA 2.9 (1.8 – 4.1) NH 2.4 (1.8 – 3.7) RI 3.2 (1.6 – 4.5); Breast cancer MA 10.9 (5.3 – 16.4) NH 9.8 (5.4 – 14.8) RI 10.7 (7.2 – 13.9); Prostate cancer MA 13.8 (7.4 – 24.0) NH 11.5 (5.9 – 17.3) RI 13.8 (9.5 – 17.7). NH has the lowest rates on all four types of cancer; MA and NH were similar regarding average rates, but MA communities had the widest disparities for lung, breast and prostate cancer. Findings suggest within and between state variations in cancer rates. Policies and programs may target geographic areas/communities with high rates of cancers, examine environmental effects on cancer rates and develop strategies in reducing cancer rates.

